# Quality of Publication Ethics in Instructions to Authors

**Published:** 2013-09

**Authors:** Viroj Wiwanitkit

**Affiliations:** Visiting Professor, Hainan Medical University, China; Visiting Professor, Faculty of Medicine, University of Nis, Serbia; Adjunct Professor, Joseph Ayobabalola University, Nigeria

**Dear Editor,**

The recent report on the quality of publication ethics in instructions to authors is very interesting.^1^ Salamat et al.^1^ concluded that “quality of publication ethics, as instructed to the authors, can improve the quality of the journals.” Indeed, the quality of a journal depends on several factors such as referencing/citation, accuracy, editorial board member, editor-in-chief, publisher, and publishing regularity as well as the publishing ethics. In order for any journal to achieve the desired quality, the publisher, editor, and author must work all together by taking into account the said factors.

As regards authors’ contribution, it is important that the journal provide the author with relevant information on the preferred manuscript format. “Specific recommendations aiming to improve publication practice” should be included in instructions to authors. ^2^ Instructions to authors constitute an integral component of a journal; nevertheless, some journals tend to overlook this vitally important detail or fail to regularly publish it in each issue.^2^ A recent study by Jaykaran et al.^3^ in India lends further credence to the previous study and chimes in with the Salamat et al.^1^ study inasmuch as it concludes that the incompleteness of instructions to authors is common.

With respect to publication ethics, Jaykaran et al.^3^ reported that complete guidance regarding ethics was provided in only seven out of the ten journals investigated. Another report from Brazil showed a very interesting statistic in that up to 79% of the journals assessed had no recommendations on ethics in their instructions to authors.^4^ Jaykaran et al.^2^ suggested that “medical journals must upgrade their instructions to authors to include ethical requirements.”^2^

Ideally, a journal’s policies, formatting requirements, and publication standards (including ethics) should be clearly elucidated in its instructions to authors. According to Salamat et al.^1^ however, not all journals furnish their authors with clear instructions. 

The reasons for the failure of a journal to provide appropriate instructions should be further investigated. Some journals might overlook the importance of instructions to authors and omit to publish this information due to page constraints or publication costs. It should be noted that any indexing and accrediting body assesses the quality of a journal’s instructions to authors when rating the quality of that publication.

In regard to ethical contraventions , the usual excuse cited is lack of knowledge and resultant confusion in consequence of insufficient instructions to authors.^5^ Some journals might assume that their authors possess the required knowledge for manuscript submission, but it would always be safer to assume that it is not possible that all authors have a good basic knowledge of publication ethics.^5^ Wager^6^ says, “Journals do not provide consistent guidance about authorship and many editors are therefore missing an important opportunity to educate potential contributors.” 

In conclusion, no journal should overlook the importance of instructions to authors. Nevertheless, the next question is how we should set out to encourage authors to read and follow these instructions. There is no doubt that the problem of scientific misconduct and breach of publishing ethics is common at present, and possible explanations might be the author’s failure to peruse the ethical standards stated in instructions to authors and the author’s failure to comply with such guidelines. More research is required to shed further light on the reasons for violations of publication ethics. 
